# Shifting sands: How parents engage with the idea of their child’s death

**DOI:** 10.1017/S1478951526103046

**Published:** 2026-06-25

**Authors:** Naomi T. Katz, Jenny L. Hynson, Lynn Gillam

**Affiliations:** 1 Victorian Paediatric Palliative Care Program, The Royal Children’s Hospital Melbourne, Melbourne, Victoria, Australia; 2 Department of Paediatrics, The University of Melbourne - Parkville Campus, Melbourne, Victoria, Australia; 3 Clinical Paediatrics Group, Murdoch Children’s Research Group, Melbourne, Victoria, Australia; 4 Children’s Bioethics Centre, The Royal Children’s Hospital Melbourne, Melbourne, Victoria, Australia

**Keywords:** Prognostic awareness, pediatric palliative care, serious illness, death, bereaved parents

## Abstract

**Objectives.:**

While parental prognostic awareness is a vital concept, its complexity can sometimes be underestimated. Our study aimed to understand how parents of children with serious illness engage with the idea of their child’s death from a lived experience perspective.

**Methods.:**

Bereaved parents of children known to a statewide pediatric palliative care service, who died at the age of 16 years or younger from any medical condition, participated in semi-structured interviews. Reflexive thematic analysis was employed, informed by a phenomenological framework.

**Results.:**

Twenty bereaved parents (6 fathers) participated; children ranged in age from 1 day to 16 years. Findings were categorized into 2 main themes: (1) Daily realities of engaging with the idea of death, with subthemes: variability in contemplating death, conversations with their child, conversations with family and friends, conversations with clinicians, making treatment decisions, and not engaging with death, and (2) factors influencing engagement, with subthemes: implicit factors, changes in clinical condition, and clinician communication.

**Significance of results.:**

Parents’ engagement with the idea of their child’s death was individual and dynamic. Our findings offer tangible examples of how parents contemplate and operationalize this engagement over time, helping clinicians appreciate the complex and non-binary nature of prognostic awareness. Clinicians should explore how parents engage with the idea of their child’s death both during and outside clinical encounters over time, acknowledge their role in supporting and guiding parents, and recognize that there is no single way for parents to process the idea of their child’s death.

## Introduction

Caring for children with serious, potentially life-threatening illnesses presents numerous challenges for both families and clinicians, with one of the hardest being the death of a child. To provide effective support, clinicians must appreciate how parents engage with the idea of their child’s death over time. However, in our experience, the complexity of this engagement can sometimes be underestimated.

To anticipate or prepare for death, one must be aware that death is a possible outcome. The term “prognostic awareness,” increasingly used in the literature, refers to a person’s understanding of their illness’s likely trajectory, including life expectancy (Jackson et al. 2013; Applebaum et al. 2014; Ozdemir et al. 2023). In pediatrics, it describes parents’ understanding of their child’s prognosis (Levine et al. 2021; Kaye et al. 2021b). It is not uncommon for parents and clinicians in both cancer and non-cancer settings to have different perceptions of a child’s prognosis (Mack et al. 2007; Sung et al. 2009; Rosenberg et al. 2014; Boss et al. 2017; Morell et al. 2021; Kaye et al. 2021b).

Prognostic awareness can have impacts on care in both adult and pediatric settings; it has been linked to fewer intensive treatments near the end of life (Wolfe et al. 2000; Chen et al. 2022), a greater emphasis on symptom- and comfort-focused treatments (Kaye et al. 2025; Kentor et al. 2025), and increased engagement with palliative care services (Nyborn et al. 2016; Mack et al. 2018). Studies have described how an accurate understanding of prognosis can guide decisions that align with goals and values, including decisions made by a parent for their child (Mack et al. 2007; Jackson et al. 2013; Rosenberg et al. 2014).

Studies also show that most parents and young people express a desire for honest diagnostic and prognostic information (Mack et al. 2006, 2018) repeated at different time points (Kaye et al. 2025; Kentor et al. 2025), even if the information is difficult or distressing to hear (Nyborn et al. 2016). However, in our experience, the way in which parents engage with the idea of their child’s death is more complex and fluid than these studies suggest. For example, parents might be willing to discuss their child’s death in one appointment but not in later appointments. This can make it challenging for clinicians to operationalize communication with parents and establish a shared prognostic awareness.

Prognostic awareness is now often viewed as multidimensional and dynamic, with cognitive and emotional components that influence decisions and actions, such as discussions about death or treatment choices (Brenner et al. 2022; Amonoo et al. 2025). However, the way this complexity appears in clinical practice, especially in pediatrics, remains incompletely described, and in our experience, parents may be assessed as either “understanding” or “not understanding” the prospect of their child’s death.

Deeper insight into how parents engage with the idea of their child’s death and factors influencing engagement has the potential to assist clinicians in better understanding parents and tailoring conversations and support, ultimately benefiting children, families, and clinicians. The aim of this study was to explore parents’ lived experience of thinking and talking about their child’s possible death.

## Methods

### Study setting and team

The study is part of the primary author’s doctoral research, conducted within a statewide pediatric palliative care program. The research team comprised palliative care physicians and a bioethicist. Institutional ethics approval was received.

### Sample and recruitment

Inclusion criteria were: English-speaking parents of a child known to the statewide pediatric palliative care service, who had died at age 16 years or younger, with the death from any medical condition occurring at least 6 months prior to, but within 10 years of, recruitment. Families were not contacted if contact was considered by palliative care staff to be potentially detrimental to their well-being.

Purposive sampling was used to ensure a range of ages at death, from cancer and non-cancer conditions. Invitations were sent by mail or email. Parents of 102 children were invited to participate. Where appropriate, both parents were invited, with the option to be interviewed together or separately.

### Data collection

A semi-structured interview schedule was used and refined iteratively. Interviews were conversational, with the interviewer following participants’ lead to elaborate on their lived experiences. Interviews lasted between 56 and 101 minutes (mean of 73). Interviews were conducted via Zoom. Audio files of each interview were saved, transcribed verbatim, and anonymized.

### Data analysis

Transcripts were managed using NVivo and Word software. The first author coded all transcripts, and a selection of transcripts was co-coded by all 3 investigators, with discussions at regular intervals to compare and consider interpretations of the data. Data were analyzed using Braun and Clarke’s 6-step reflexive thematic analysis (RTA), offering a flexible yet rigorous approach to theme generation (Braun and Clarke 2021). A phenomenological framework underpinned the study, with the analysis grounded in parents’ lived experience (Ayton et al. 2023). Analysis was iterative and inductive, with the investigators’ clinical experience closely linked to and contributing to the data analysis. Reflexive engagement throughout the process was employed.

## Results

Twenty parents of 15 children participated; 6 (30%) were fathers. Seven (47%) children had a cancer diagnosis (Table 1). Two sets of parents participated in separate interviews, and 3 sets participated jointly in one interview (Table 2).

**Table 1.** Parent and child details (M denotes mother, F denotes father) Table 1 long description.

Parent	Time since bereavement (months)	Child age	Cancer
M1	29	8 years	Yes
M2	19	5 years	No
M3	47	8 months	No
F4			
M5	15	9 years	Yes
M6	22	5 years	No
M7	23	1 day (antenatal diagnosis)	No
M8	22	13 months	No
F9			
M10	39	4 years	No
F11	40		
M12	11	1 year	Yes
M13	23	2 years	No
M14	77	16 years	Yes
M15	56	4 years	Yes
F16			
M17	16	12 years	Yes
F18			
F19	65	9 years	Yes
M20	49	9 years	No

**Table 2.** Themes, subthemes, and illustrative quotes Table 2 long description.

Theme	Subtheme	Sample quotes
The daily realities of engaging with the idea of death	Variability in contemplating the idea of death	…*this is the treatment plan and if everything goes smoothly… then she would have a one in three chance of survival…*
		*…there was a season there*… *we actually didn’t talk about the fact that she was going to die because she was good!* (M12, cancer)
		*We have conversation over conversation. But it’s different when the time’s come. No, nothing can prepare. Just like the lady, the counsellor told me, like, you’re just not able to prepare beforehand. Yeah, it’s totally different when you face it.* (M10, non-cancer)
	Conversations about death with their child	*…along the way I said… cancers are very complex… sometimes it goes away and sometimes it comes back… it’s not like you’re going to have this medicine and you’re going to be fixed because I didn’t want to paint this rainbow for him because I just felt that was unfair…* (M5, cancer)
	Conversations about death with family and friends	*…and I have good friend support, but there’s often times that I found that very frustrating as well because friends would say things that [are] completely unhelpful… (M12, cancer)*
	Conversations about death with clinicians	*…getting pediatric palliative care involved, I wasn’t ready. I didn’t want, because that meant my child was dying and I didn’t, and I wasn’t ready to go there.* (M14, cancer)
	Making treatment decisions	*…That’s not the life we wanted for him. But… if there’d been a thousand kids or ten kids, or one kid before [baby] and the treatment had not worked, then to undergo the treatment is about us not being able to make a difficult decision that is the right one for our son… But because it was untested and no one, no child had been, undertaken this treatment at that age, the diet, we went, well, we have to give him the best chance of success.* (F9, non-cancer)
		*So we say no… this is not a life for a kid… she needs to be able to go to a park, enjoy, be happy and she’s not gonna be in a hospital for later you gonna say that treatment didn’t work, because that’s what the doctor said, if this one doesn’t work then we’ll do another one…* (M1, cancer)
	Not engaging with death	*Like we were very aware that [child] was not going to live a full life, but we were never focused on him passing. We just worried about getting on with it and giving him the experiences that he needed to have.* (F9, non-cancer)
Factors influencing engagement with the idea of death	Implicit factors	*And the second time when he told us (relapse)… we kind of assumed she’s gonna go, we just knew because we learned you know with the time, to understand, to see…* (M1, cancer)
	Changing clinical condition	*And then from that time onward we still have a hope of her being cured… But at the end, we found like she’s way more serious than that. After 2 years old… then we knew that it’s really, no hope for her to stay with us for a long time…* (M10, non-cancer)
	Clinician communication	*And I think because [oncologist] was matter of fact at the beginning, and explained the nature of the tumor, why it was inoperable, and why it was incurable, I guess just made us focus on the present moment with [child].* (M15, cancer)
		*…and it’s almost like you know, you’d ask the question and I think a conversation for me would have been helpful to be the reality of this situation is, to be a bit more factual… Like, you know we will try everything we can but sorry, you know…* (M5, cancer)

Findings fell into 2 broad themes: the daily realities of parents engaging with the idea of their child’s death, and factors that influenced engagement. Themes and subthemes are presented below, along with a summary table that includes additional illustrative quotes (Table 2).

### Daily realities of engaging with the idea of death

Parents described engaging with the idea of death in various ways that fluctuated over time and across contexts. These included how often they contemplated death, who they discussed it with, how it informed decisions, and when they chose not to engage at all.

#### Variability in contemplating the idea of death

Engagement with the idea of death occurred at different times between parents and also changed over time for individual parents. Contemplation of death soon after diagnosis was seen, “*So when I went to hospital, I remember I said, my daughter is gonna die. I said to my husband, she’s gonna die…”* (M1, cancer)

Conversely, engagement with the idea of death only when their child was near the end of their life was also seen, “*…when I saw how she was going, how she was shutting down… that’s when I gave in to the horrible disease, knowing that it was going to take our daughter.”* (F18, cancer)

Even parents who contemplated death early experienced changes in their thinking over time, and did not simply “always” or “never” contemplate death. One parent described both a pervasive background worry, “*Yeah, and I think you know, there was always that little voice in the back of my mind, you know that he might not survive this,”* and how engaging with death fluctuated over the course of the illness trajectory, “*…in the first line of treatment, you think he’s going to get through this… and I suppose when the first relapse happens… you have this little bit of doubt.”* (M5, cancer)

Another parent shared how their thinking changed over time.

… it was really actually only the 24 hours that we probably completely… this is it… I think we just really needed that time… until then, we were still, as much as it was… very grim and not good… we were waiting on blood tests to come back to say that the markers are changing and the medicines were working… (M12, cancer)

Importantly, contemplating the inevitability of death did not necessarily mean being ready or wanting to talk about it, “*It’s hard to say out loud, because maybe, I hope it wasn’t real. But I look back, I knew that that was right.* (M10, cancer)

Somewhat predictable deaths came as a surprise to parents when children had previously recovered from being unwell.

But then his actual passing was quite by surprise. We spoke to the attending consultant… she said… it’s not looking great, but this is [child], so I think he will pull through, not medically, but just because he does that. Which was fair. But he didn’t. (F4, non-cancer)

Finally, even parents who understood that their child would ultimately die did not always emotionally or psychologically engage with this, *“…so I think by that point, I still didn’t want to accept that it wasn’t going to be long, but I think I knew.”* (M15, cancer)

#### Conversations about death with their child

Parents’ engagement with the idea of their child’s death was reflected in their various approaches to discussing death with their seriously ill child. One mother sought to provide honesty while avoiding the word death, as her son did not like this word (Table 2). Another mother wanted her child to have permission to die, “*…I always talked to her when she was sleeping and tell her, ‘you can go, you can go, I will be fine, I promise, I will be fine’…”* (M1, cancer)

The potential for both parents of the same child to engage differently in conversations was highlighted.

…[mother] was very adamant that… [child] doesn’t know that he’s got a terminal illness… I did support it but would have liked more time… so that whatever the decision, to have a bit more confidence, because now it’s what burns me quite a bit, that I was dishonest to [child].” (F19, cancer)

#### Conversations about death with family and friends

Speaking with family and friends was another context in which engagement with the idea of death could be seen. Some parents highlighted the value of a shared understanding with parents of children with a similar condition, “*When you meet people in Day Oncology or even in the wards, you just have to look at each other and you get it.”* (M17, cancer)

Preparing their family for the possibility of death was described, *“…I would talk about, you know, chances of him passing away… I would be very clear to my parents… just to prepare them…”* (M3, non-cancer)

Conversely, some parents elected not to talk with family or friends, “…*we relied heavily on our family’s excitement for [child] to keep me going, because while I was in my family, I was having a normal pregnancy that I got to just enjoy my son.”* (M7, non-cancer)

#### Conversations about death with clinicians

Engagement with the idea of death was also reflected in conversations with clinicians. Some parents wanted early advance care planning conversations, “*I had instigated asking for palliative care… and putting an advance care plan in place… do we actually have to keep getting on this merry-go-round where we’re just watching him get sicker and sicker?”* (M6, non-cancer)

Other parents engaged in advance care planning conversations instigated by clinicians, *“…so we had that conversation about the ‘do not resuscitate’… she explained… if they do resuscitate and then it’s tubes and… being kept alive…”* (M5, cancer)

One parent described not feeling ready to contemplate her child’s death during the conversation where she was told about her son’s disease progression and incurability (Table 2).

The balance between the value and burden of prognostic conversations, along with the possibility that both parents of the same child may have different needs, was described. One father shared, “*…I need to know, I want to know everything. It doesn’t mean it’s all going to happen”* (F16, cancer), while his wife shared, “*…you need to be prepared… but once you know, there’s no not knowing. And just… it was really hard to accept that [child] might experience some of those symptoms…”* (M15, cancer)

Finally, not engaging with the idea of death did not mean not engaging in advance care planning conversations. One mother reflected the importance of revisiting discussions with changing clinical circumstances rather than clinicians making assumptions, *“…his medical issues are different. And our stage in life would be different. So yeah, I mean, our answer would have always been the same, but I don’t think that can be assumed.”* (M20, non-cancer)

#### Making treatment decisions

Engagement with the idea of death was also expressed in the concrete decisions parents made. Some parents shifted from life-sustaining treatments to focusing on symptoms and comfort as they recognized the inevitability of their child’s condition worsening, *“…he had basically a tube coming out of every orifice in his body, coming and going from everywhere. So, we’re going to put him back in that place and I couldn’t do that to him.”* (F19, cancer)

For some parents, the thought of death prompted the pursuit of every available treatment option.

…you’d try anything… you want to believe that you did everything in your possible power to help your child. Because if you didn’t… you failed… I took questions… I took a little vial in and said, this is what I’m going to give her… (M17, cancer)

Crucially, understanding the prospect of their child’s death and prioritizing quality over quantity of life did not necessarily mean parents chose to limit life-sustaining treatments (Table 2).

…they said to us, we don’t think he’s going to make it. And we responded… yes, we understand the situation is really dire… that’s understood. But we’re also kind of trying things, trying to find out what it is, to try to help him get out of it.” (F4, non-cancer)

#### Not engaging with death

Some parents described a focus on life rather than death, in terms of both survival and living in the present. One father described how even a small probability of a cure was enough for him to focus on his daughter’s survival, not death.

I thought modern medicine was going to be good enough to cure our daughter, I really, truly believed that they were going to beat this because there was a percentage of hope… whether it was 5%, 10%, for me it was 100%. (F18, cancer)

Other parents described an active decision to focus on day-to-day life and making memories, “…*I guess it was the hope for his experience, because we always knew what the outcome would be.”* (M15, cancer)

[father] and I just could see where we were going… we’d started to talk about making memories and making them fast and doing the things that we wanted to do ‘cause we didn’t have time up our sleeve. (M6, non-cancer)

### Factors influencing engagement with the idea of death

Parents’ engagement with their child’s death was shaped by both implicit factors and external influences, including their child’s clinical condition and how clinicians communicated with them.

#### Implicit factors

Some parents had an intuition about their child’s impending death, “*…my mother’s instinct, I know that she is, she won’t have much time. It’s just something very strange. I know that it’s coming soon.”* (M10, non-cancer)

…we felt was his last Christmas, and it did end up being his last Christmas.” (M6, non-cancer)

#### Changing clinical condition

Parents’ engagement with the idea of death was shaped by stability and instability in their child’s medical condition. Seeing their child unwell, or obtaining more clinical information, led to contemplation of the possibility of death.

…she had the bone marrow transplant… then the stoma… she needed that done, and then redone. And she had TPN for a while… there were times in that one year admission that I didn’t know if I’d bring her home. (M2, non-cancer)

Conversely, periods of clinical stability or improvement, especially when this went against what had been anticipated, distanced parents from engaging with the idea of death.

…every time he made it through… I always kind of pushed that a little bit further out and just thought, he could potentially survive another year, or another year. (M8, non-cancer)

Besides watching their own child’s progress, comparing their child’s condition with that of other children prompted some parents to realize the inevitability of their child’s death.

…I looked at the other two boys… and they were still walking independently and eating independently and talking. And I looked at [child]… and I realized we didn’t have as much time. (M6, non-cancer)

#### Clinician communication

Various aspects of communication with clinicians influenced how parents engaged with the idea of their child’s death.

Clear clinician communication led some parents to understand the inevitability of death, *“I think it was very clear, I came out of that meeting very clear that [child] is not going to survive.”* (F19, cancer)

Conversely, ambiguous language or mixed messages about prognosis were confusing, “*…if you tell me what they told me, she has an aggressive cancer, how should I know what an aggressive cancer is?”* (M1, cancer)

…he kind of spoke in terms of, ‘we’re going to do things to minimize the long-term impact’, like the prognosis was different… it might give you some hope when the prognosis had not changed.” (M15, cancer)

Parents could interpret the same discussion about prognosis differently.

I had a very different understanding of the conversation to what [father] did… “incurable and lifelong treatment”… I remember… [father] going, I wonder how that will impact his job in the future, having to have these treatments… I was actually thinking that I’m going to lose him really, really soon. (M6, non-cancer)

Focus on disease-targeted treatments could cause confusion when parents were already contemplating their child’s death, *“…and then the doctors tell you… this is gonna happen, we gonna do this… and then you doubt yourself and you think okay, I’m being the pessimistic here… And then you keep going…”* (M1, cancer)

Humanity and compassion from clinicians helped parents to engage in conversations about their child’s condition and future.

… and he used his name. And then for the first time, I didn’t feel like I was just a number and that I was just someone that they were trying to say, well, make a decision and we need to see someone else next. (M7, non-cancer)

Parents appreciated having time to consider the bigger picture and treatment options, rather than concentrating only on death.

It’s how they deliver these kind of things, and give us the choices, or give us time to think about it and not just jump into… where they’re in that clinical mindset, where they’d be like, right he’s not well, he’s not breathing, his brain is dead, that’s it. (M8, non-cancer)

## Discussion

While existing literature emphasizes the multidimensional and dynamic nature of prognostic awareness and its benefits, in our clinical practice, we still observe clinicians categorizing parents as either “getting” or “not getting” the idea of their child’s death. Our study adds to the literature by describing how, first, parents varied in the timing and extent to which they contemplated the idea of their child’s death, and second, in how they engaged with and operationalized such thoughts. Contemplation and engagement with the idea of death were influenced by implicit and external factors.

Understanding the complex, individual, and dynamic nature of how parents engage with the idea of their child’s death is important because assumptions about parents’ experiences can lead clinicians to miscalculate conversations and efforts to support parents. Among other consequences, such a mismatch risks fracturing the therapeutic alliance, failing to address parents’ informational or emotional needs, and contributing to treatment decisions that may not align with a child’s best interests (Katz et al. 2021).

In our study, clinical information and how clinicians communicated that information influenced not only parents’ contemplation of death but also their readiness to engage in conversations and planning for the possibility of death. This underscores the dynamic nature of both cognitive engagement and operationalization of this information. Our findings also indicate the potential for a disconnect between cognitive understanding and emotional acceptance of death, which has been similarly described in a study of bereaved parents whose emotional acceptance of their child’s death occurred later than their intellectual understanding (Valdimarsdóttir et al. 2007).

The influence of a changing clinical picture on parents’ understanding of prognosis is also reflected in the literature (Arcadi et al. 2025). Bogetz et al. (2020) described how parents of children with chronic illness expected recovery when their child had previously survived near-death episodes. This reflects the inherent challenge of prognostication, particularly in pediatrics (Bergstraesser et al. 2021), making it hard to determine a definitive “prognostic truth” at any given moment. It is therefore understandable that parents’ contemplation of and engagement with the possibility of their child’s death is not fixed, as observed in our study.

Our study demonstrates how clinicians can both facilitate and impede parents’ engagement with the idea of their child’s death. Effective communication methods included clear language and consistent messaging, using the child’s name, addressing parents’ perceptions of the situation, and acknowledging that some parents may need to consider treatment limitations as one, but not necessarily the only, approach for their child. Clinician influence on prognostic awareness is evident in both adult and pediatric literature, as well as in ongoing efforts to enhance clinician communication and strengthen the therapeutic relationship between parents and clinicians (Kaye et al. 2021a, 2021b; Porter et al. 2022, 2023; Zalud et al. 2024; Arcadi et al. 2025).

Between parents, a spectrum of engagement with or operationalizing the idea of death was seen, from initiating advance care planning discussions to pursuing disease-targeted treatments. This is important because, while clinicians may avoid discussions about prognosis or death for fear of causing distress and taking away hope (Mack et al. 2007), parents may already be contemplating and wanting to talk about these concerns. As seen in our study, failing to recognize parents’ desire or readiness to consider their child’s death can leave parents questioning or feeling isolated in their own assessment of their child’s condition.

Our findings show what might seem paradoxical to clinicians. Parents who understood that their child would not be cured or that death was inevitable did not necessarily want to discuss this. Similarly, prioritizing quality of life over quantity did not always lead to decisions to stop life-sustaining treatments; in other words, prognostic awareness did not necessarily result in fewer intensive interventions, contrary to what has been described in the literature (Wolfe et al. 2000; Chen et al. 2022). Moreover, not believing that their child would die did not mean parents could not engage in advance care planning conversations. These examples illustrate the complexity of how parents engage with the idea of their child’s death and mirror what we see in our clinical experience. For instance, we see parents who have end-of-life plans in place and are simultaneously seeking alternative treatments, and parents who are hesitant to discuss their child’s death with clinicians while also planning their funeral. These scenarios highlight how aspects of engagement with the idea of death can be invisible to clinicians, making it hard to fully understand how parents are processing their child’s death or translating that understanding into action. This underscores the importance of avoiding assumptions and dedicating time to understanding how parents are engaging with their child’s condition at different points. For example, asking parents what they are thinking, worried about, or discussing with their child, family, or friends can provide valuable insights and help guide conversations.

## Strength and limitations

The study’s use of RTA within a phenomenological framework allowed close attention to parents’ lived experiences and meaning-making across diverse diagnoses and clinical trajectories. However, including only English-speaking parents may limit the transferability of the findings to culturally and linguistically diverse groups. Second, the study is susceptible to selection bias and retrospective methodology; parents who chose to take part may differ from non-respondents in how they have processed, and continue to process, their child’s illness and death over time.

## Conclusions

Our study offers clinicians who do not often encounter childhood death tangible examples of the individual, dynamic, and complex ways parents both contemplate and engage with the idea of their child’s death. Understanding this can help clinicians avoid seeing prognostic awareness as simply present or absent and prevent assumptions about parents’ thoughts or readiness for conversations, especially during a single encounter, ultimately translating into benefits for the child, parents, and clinicians. Clinicians should take the time to explore how parents are engaging with the idea of their child’s death, both in and outside clinical settings over time. Finally, clinicians should recognize their role in guiding and supporting parents, acknowledging that there is no one-size-fits-all approach for parents to engage with the idea of their child’s death.

## Table 1 Long description

The table lists individual parents identified as mother or father, with time since bereavement in months, the child’s age, and whether cancer was reported. Most rows are mothers; several fathers are listed but many father entries have missing details beyond the parent identifier. Where provided, time since bereavement ranges from 11 months to 77 months, with many clustered in the teens to forties. Child ages span from 1 day (noted as antenatal diagnosis) and 8 months through 16 years, with several children aged 4 to 9 years. Cancer status is recorded as Yes for multiple mothers and one father, and No for several mothers; some rows have cancer status missing. Missing data are present for F4, F9, F11 (missing child age and cancer), F16, and F18, so counts and comparisons by parent type are incomplete.

Navigate back to Table 1.

## Table 2 Long description

The table organizes qualitative findings into two themes, each with subthemes and illustrative parent quotes about engaging with the idea of a child’s death. Theme one covers daily realities, including variability in thinking about death, talking about death with the child, family and friends, and clinicians, making treatment decisions, and sometimes avoiding focus on death. Quotes highlight uncertainty about prognosis, difficulty preparing until the situation becomes immediate, and frustration with unhelpful comments from friends. Communication with clinicians is portrayed as pivotal, with palliative care discussions sometimes experienced as confronting and hard to accept. Treatment decision quotes contrast pursuing untested options to give a child the best chance with refusing burdensome care seen as not offering a meaningful life. Theme two describes factors influencing engagement, including implicit cues, deterioration in the child’s condition, and clinician communication style. Across subthemes, engagement tends to intensify as relapse or worsening condition makes outcomes feel more certain, and clearer, more factual clinician explanations can shift families toward focusing on the present. The quotes are illustrative examples from participants and are not intended to represent frequency or prevalence of views.

Navigate back to Table 2.
